# Identification and Analysis of the *MIR399* Gene Family in Grapevine Reveal Their Potential Functions in Abiotic Stress

**DOI:** 10.3390/ijms25052979

**Published:** 2024-03-04

**Authors:** Jingjing Liu, Yi Ren, Yan Sun, Yonggang Yin, Bin Han, Lipeng Zhang, Yue Song, Zhen Zhang, Yuanyuan Xu, Dongying Fan, Junpeng Li, Huaifeng Liu, Chao Ma

**Affiliations:** 1 Key Laboratory of Special Fruits and Vegetables Cultivation Physiology and Germplasm Resources Utilization of Xinjiang Production and Construction Corps, Department of Horticulture, Agricultural College of Shihezi University, Shihezi 832003, China; jingjing@stu.shzu.edu.cn (J.L.);; 2 College of Landscape and Horticulture, Yunnan Agricultural University, Kunming 650201, China; 3 Changli Research Institute of Fruit Trees, Hebei Academy of Agricultural and Forestry Sciences, Changli 066600, China; 4 Shanghai Collaborative Innovation Center of Agri-Seeds, School of Agriculture and Biology, Shanghai Jiao Tong University, Shanghai 200240, China

**Keywords:** *Vvi-MIR399*, grapevine, abiotic stress, evolutionary conservation, potential functions, expression pattern

## Abstract

MiR399 plays an important role in plant growth and development. The objective of the present study was to elucidate the evolutionary characteristics of the *MIR399* gene family in grapevine and investigate its role in stress response. To comprehensively investigate the functions of miR399 in grapevine, nine members of the *Vvi-MIR399* family were identified based on the genome, using a miRBase database search, located on four chromosomes (Chr 2, Chr 10, Chr 15, and Chr 16). The lengths of the Vvi-miR399 precursor sequences ranged from 82 to 122 nt and they formed stable stem–loop structures, indicating that they could produce microRNAs (miRNAs). Furthermore, our results suggested that the 2 to 20 nt region of miR399 mature sequences were relatively conserved among family members. Phylogenetic analysis revealed that the *Vvi-MIR399 members* of dicots (*Arabidopsis*, tomato, and sweet orange) and monocots (rice and grapevine) could be divided into three clades, and most of the *Vvi-MIR399s* were closely related to sweet orange in dicots. Promoter analysis of *Vvi-MIR399s* showed that the majority of the predicted *cis*-elements were related to stress response. A total of 66.7% (6/9) of the *Vvi-MIR399* promoters harbored drought, GA, and SA response elements, and 44.4% (4/9) of the *Vvi-MIRR399* promoters also presented elements involved in ABA and MeJA response. The expression trend of *Vvi-MIR399s* was consistent in different tissues, with the lowest expression level in mature and young fruits and the highest expression level in stems and young leaves. However, nine *Vvi-MIR399s* and four target genes showed different expression patterns when exposed to low light, high light, heat, cold, drought, and salt stress. Interestingly, a putative target of *Vvi-MIR399* targeted multiple genes; for example, seven *Vvi-MIR399s* simultaneously targeted *VIT_213s0067g03280.1*. Furthermore, overexpression of *Vvi_MIR399e* and *Vvi_MIR399f* in *Arabidopsis* enhanced tolerance to drought compared with wild-type (WT). In contrast, the survival rate of *Vvi_MIR399d*-overexpressed plants were zero after drought stress. In conclusion, *Vvi-MIR399e* and *Vvi-MIR399f*, which are related to drought tolerance in grapevine, provide candidate genes for future drought resistance breeding.

## 1. Introduction

MicroRNAs (miRNAs) are a class of endogenous, single-stranded, small regulatory RNA molecules, which widely exist in various eukaryotes [1]. In plants, miRNAs range in length from 20 to 24 nt and are processed from longer precursor miRNA (pre-miRNA) molecules with stem–loop structures [2,3]. The pre-miRNA is processed by *DICER LIKE1* (*DCL1*) into mature miRNA, which then regulates gene expression by binding mRNAs and inducing degradation or translation repression [4,5]. Thus, plant miRNAs are thought to mediate almost all plant cellular and metabolic processes via regulating post-transcriptional gene silencing [6,7]. In plants, miRNAs play an important role in a number of physiological processes, such as growth development and biotic and abiotic stress responses, where they can regulate the expression levels of their target genes by cleavage.

With the advent of sequencing technology, multiple miRNAs have been identified in various species, tissues, and physiological conditions [8,9], and they participate in the whole process of plant growth and development. The regulatory module of *hpe-miR162a_L-2-ARF19* is involved in early seed development [10]. In *Medicago truncatula*, overexpression of *MtMIR166-insensitive REVOLUTA* (*MtREV1*) led to adaxialized leaves and ectopic leaflets along the dorsoventral axis [11]. The crown root defect 1 (*CRD1*) regulates crown root development via the miR156 pathway [12]. Rice-specific osa-miR5506 plays an essential role in the regulation of spikelet determinacy, floral organ number, and female gametophyte development [13]. Furthermore, cme-miR2 may modulate the cell growth and fruit size of Hami melon by negatively regulating *TIP GROWTH DEFECTIVE 1* (*TIP1*) [14]. Apple fruit was mostly derived from the hypanthium contributed mostly by sepal tissues, which were positively regulated by *APETALA2* (*AP2*) and over-accumulation of miR172 leading to the silencing of *AP2*, then leading to dramatic reductions in fruit size and weight [15]. The *CsmiR396-CsGRFs*/*CsGIFs* module is diverse in cucumber, and *CsGRF3* and *CsGRF5* play opposite roles in regulating cell proliferation [16]. The miR156 of tomato targeted *SPL*/*SBP* box transcription factors involved in maintenance of the meristematic state of ovary tissues, thereby controlling the initial steps of fleshy fruit development [17]. *SlMIR164b* is required for flower and shoot boundary specification, and *SlMIR164a* is required for fruit growth including the expansion of its outer epidermis, which determines the properties of the fruit skin of tomato [18]. Mul-miR477 can repress the expression of the antisense long non-coding RNA (*Mul-ABCB19AS*) in mulberry and increase the expression of the ATP binding cassette (ABC) transporter B 19 gene (*Mul-ABCB19*), and it acts as a positive regulator participating in anthocyanin accumulation through the regulatory network of *miR477*-, *Mul-ABCB19AS*-, and *Mul-ABCB19* [19]. All the above-mentioned studies indicated that miRNAs have extensive regulatory functions in plant growth and development.

Some evidence in plants indicates that the miRNA family might play crucial roles in responses to abiotic and biotic stresses. For example, the miR172-*IDS1* module is critically involved in the reactive oxygen species (ROS) scavenging and re-establishment of redox homeostasis in high salinity environments, which further contributes to rice salt tolerance [20]. In maize, the *GRMZM2G427404* (*CRP04*) transcript is targeted by miR190 and may encode a protein that positively regulates drought stress tolerance via an abscisic acid-dependent pathway [21]. Mulberry miR166f targets the transcripts encoding a homeobox-leucine zipper transcription factor whose expression is induced by drought stress and is a positive regulator of drought stress tolerance [22]. Knockout of miR482b and miR482c elicit the expression perturbation of other miRNAs in tomato, suggesting a novel mechanism with cross-regulation of miRNA in tomato [23]. After cotton was infected with *Verticillium dahliae*, the expression of microRNA 166 (miR166) and miR159 was increased and exported to fungal hyphae for specific silencing [24]. Moreover, miR159 was found to be abundant in *Arabidopsis* galls after inoculation of root-knot nematodes, and the miR159abc triple mutant was more resistant to root-knot nematodes [25]. Therefore, to more comprehensively recognize the roles of a miRNA family with multiple members, it is necessary and significant to carry out a systematic analysis of each member.

Many plant miRNAs are evolutionarily conserved between species and can be divided into different families that are located at one or more genomic loci [26]. In the miRNA family, even though precursors of different members produce the same or similar mature miRNAs, they exhibit different spatial and temporal expression profiles, resulting in functional diversification that leads to the regulation of different target genes [27]. In recent years, miR399 has been shown to play an important role in plant phosphorus signaling pathways [28]. MiR399s are highly conserved in plants and have been identified in more than 30 species of plants according to the miRBase database [29]. Among them, The *Arabidopsis* miR399 family contains 7 genes, rice has 11 members, tomato has 2 members, and grapevine (*Vitis vinifera* L.) has 9 members. It is generally accepted that conserved miRNAs might play crucial roles in regulating fundamentally important biological processes [30].

MiR399 was the first miRNA demonstrated to have the ability to increase plant uptake of phosphorus (P) [31,32,33]. MiR399 has been proposed to be conserved in angiosperms in response to P starvation [34,35,36]. Its target has been identified as *UBC24*, which encodes a ubiquitin-conjugating E2 enzyme, also known as *PHOSPHATE 2* (*PHO2* or *UBC24*) [37]. Meanwhile, miR399 might improve phosphorus absorption and metabolism in rice and *Arabidopsis* by reducing the transcription level of *PHO2*. *CsUBC24* downregulates the SEPALLATA family, which disrupts the floral meristem identity regulatory network and leads to developmental abnormalities in flowers. By interacting with INDUCER OF CBF EXPRESSION1 (*CsICE1*), *CsUBC24* disturbs stomate function on the anther surface, which inhibits anther dehiscence [38]. In transgenic plants, increasing miR399 transcripts reduces *UBC24* transcripts [39]. Under freezing stress, overexpression of tae-miR399 ultimately decreases the expression of *AtUBC24*, inhibiting the degradation of *ICE1* (Inducer of *CBF* expression 1), which increases the expression of genes involved in the *C-REPEAT BINDING FACTOR* (*CBF*) signaling pathway [40]. Although *UBC24* was the target of miR399, we did not find literature on the association between UBC24 and specific processes or traits in grapevine.

Grapevine is widely planted worldwide and can be used as a variety for the production of table grapes, juice, and wine. Currently, little information is available on the relationship of miR399 in grapevine. To understand the function of this important miRNA family in grapevine, we conducted a comprehensive analysis to identify *Vvi-MIR399* family members. In this study, multiple bioinformatic tools and online websites were combined to identify the *Vvi-MIR399* family members of grapevine. Moreover, the *cis*-elements in the promoter of the *Vvi-MIR399* gene family and the target genes predicting the *Vvi-MIR399* family were also explored. In the future, miRNAs and their predicted target genes could be used in molecular breeding strategies for disease-resistant or stress-tolerant varieties, offering meaningful views and methods for developing the grapevine industry.

## 2. Results

### 2.1. Distribution of miR399 Family Members in Grapevine

**Figure 1:**
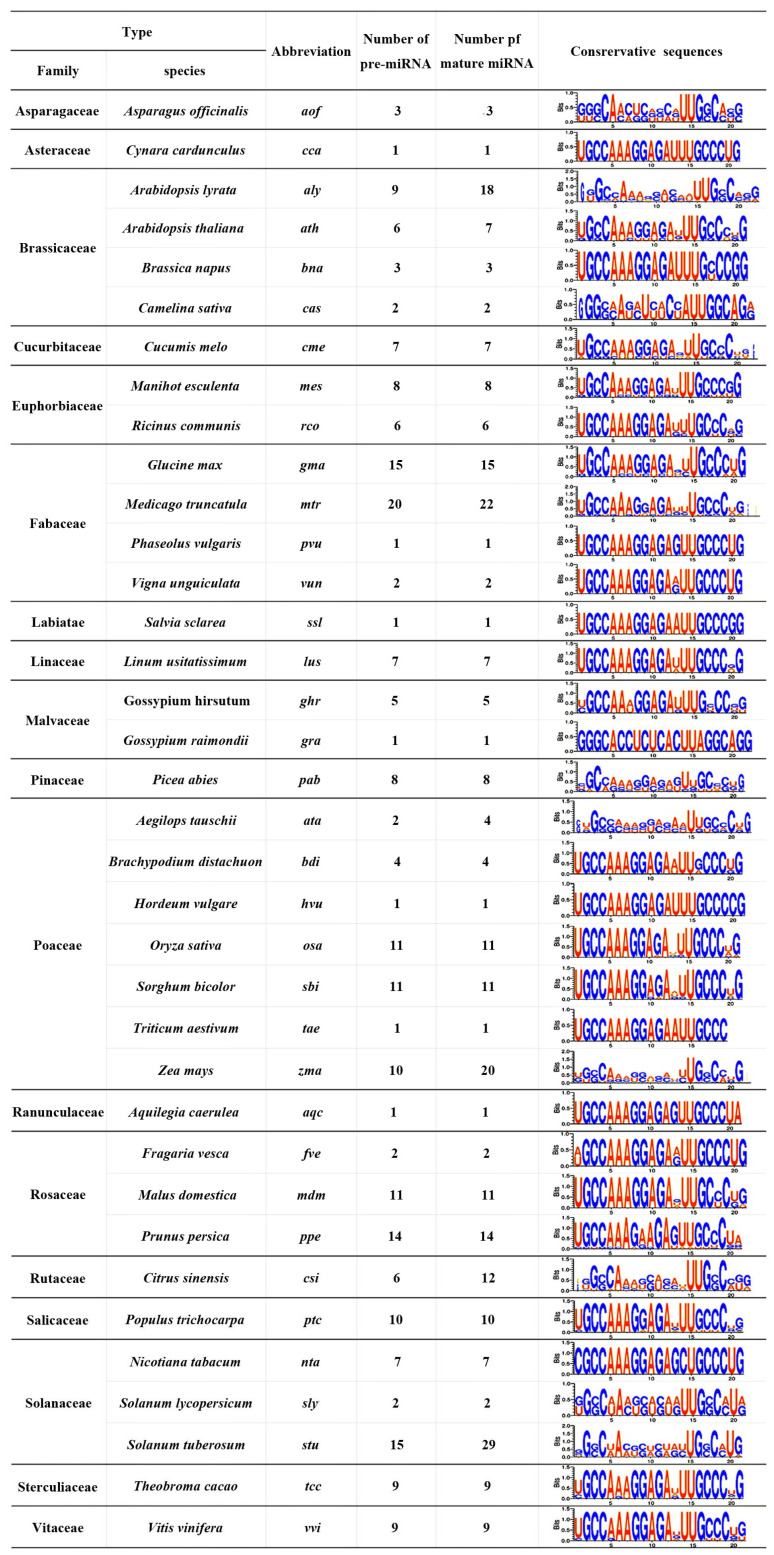
Distribution and quantity of plant miR399 family members.

The miR399 precursor and mature sequences in all plants were searched for using the miRBase database. A total of 231 precursor miR399 and 275 mature miR399 sequences from 36 species and 18 families were obtained. Among them, there were six, two, eleven, six, and nine members of the precursor miR399 gene family of *Arabidopsis*, tomato, rice, sweet orange, and grapevine, respectively (Figure 1). Sequences of nine mature miR399 families were also obtained from grapevine. The lengths of the precursor and mature sequences of the Vvi-miR399 gene family were 82~122 nt and 21 nt, respectively (Supplementary Table S1). Nine members of the *MIR399* gene family were verified by cloning and sequencing using the grapevine as a cDNA template (Supplementary Figure S1). These results suggested that the number of mature sequences of miR399 in a species was not equal to the number of its corresponding precursors, and different mature sequences might come from the same precursor or multiple precursors. The number of miR399 family members and their precursors was also different in different plants, indicating a high degree of evolutionary complexity in the plant miR399 family.

### 2.2. Analysis of the Vvi-miR399 Family Precursor and Mature Sequences

**Figure 2:**
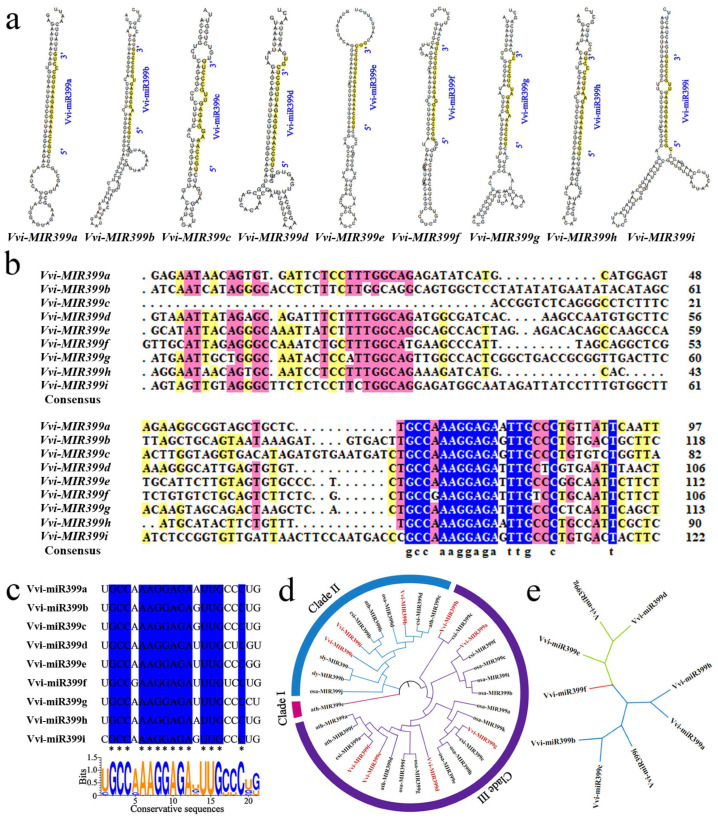
Analysis of the Vvi-miR399 family precursor and mature sequences. (**a**) Stem–loop structures of the *Vvi-MIR399* gene family in grapevine. The mature Vvi-miR399 portions are highlighted by yellow bars. (**b**) Sequence alignment of the miR399 precursor family members in grapevine. Vvi, Vitis vinifera (grapevine); blue, 100% conservation; pink, ≥75% conservation; yellow, ≥50% conservation. (**c**) The conserved domain analysis and mature sequence alignment of the Vvi-miR399 family. Identical bases in all sequences are marked in * and dark blue. (**d**) Evolutionary relationship analysis of the *Vvi-MIR399* gene family members and the other four plants. ath, *Arabidopsis*; sly, tomato; csi, sweet orange; osa, rice; Grapevine *MIR399* members are highlighted by different colors (marked with red). The *MIR399* genes of the five plants were clustered into three clades. Pink, blue, and purple indicate clades I to III, respectively. (**e**) Phylogenetic analysis of Vvi-miR399 family members. The three clades were represented in red, blue, and green.

To confirm the secondary structures of precursors in the Vvi-miR399 family, all of the precursor sequences were predicted using the website to form a stable stem–loop structure, indicating their ability to produce miRNAs (Figure 2a). Vvi-miR399s were located on the 3′arm of the secondary stem–loop hairpin structure of the *Vvi-MIR399s*. The minimum free energy of the *Vvi-MIR399s* ranged from −55.70 kcal/mol to −37.40 kcal/mol, which indicated that the *Vvi-MIR399s* were relatively stable. The sequences of the *MIR399* gene family from grapevine were aligned, and the corresponding phylogenetic tree was constructed. We observed the highest degree of conservation along the arms of the secondary stem–loop structure, especially in the core region of the mature miRNA (GCCaAAGGAGAtTTG), with more variation flanking the mature miRNA (Figure 2b). To further investigate the conserved region in the Vvi-miR399 family, multiple alignment was conducted, and the results showed that the sequences ranging from 2 to 20 nt were relatively conserved, and base preference analysis further confirmed this observation (Figure 2c).

To understand the evolutionary differences, the phylogenetic relationships of precursors in the miR399 members derived from dicot (*Arabidopsis*, tomato, and sweet orange) and monocot (rice and grapevine) plants were analyzed. The results showed that three major clades were clustered (Figure 2d). Nearly 64.71% (22/34) of the *MIR399* gene family from the five plant species were grouped into one clade (Clade Ⅲ), signifying a common ancestry and similar evolutionary path. Most of the *Vvi-MIR399* gene family were clustered with the *MIR339* genes from dicot plants. Interestingly, *Ath-MIR399e* does not form part of any specific clade and appears to have diverged independently. Furthermore, we found that all miR399 genes were also divided into three clades in grapevine (Figure 2e). Vvi-miR399a and Vvi-miR399h were grouped together with Vvi-miR399b, Vvi-miR399c, and Vvi-miR399i, while Vvi-miR399g and Vvi-miR399d formed a branch with Vvi-miR399e. In contrast, Vvi-miR399f formed a separate branch, suggesting that the identified Vvi-miR399s might have evolutionary diversification. These results supported the evolutionary conservation and diversification of miR399 family members in grapevine.

### 2.3. Prediction of Cis-Acting Elements on the Promoters of the Vvi-MIR399 Family

**Figure 3:**
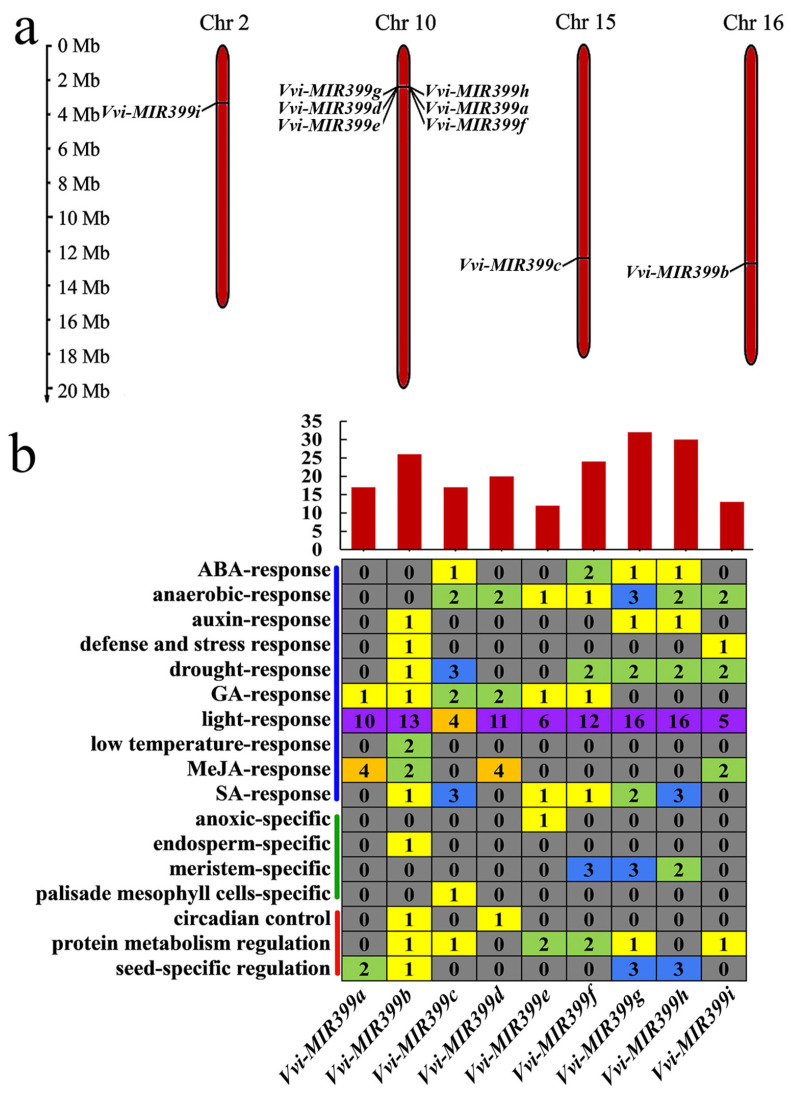
Prediction of *cis*-acting elements on the promoters of the precursor sequences of the *Vvi-MIR399* family. (**a**) Chromosomal localization of the *Vvi-MIR399* family members. The scale is in megabases (Mb). (**b**) Prediction of *cis*-acting elements on the promoters of the *Vvi-MIR399* family. The upper image shows a summary of *cis*-acting elements in the promoter region of *Vvi-MIR399* family members. The image in the middle shows the number of elements contained in the promoters of the *Vvi-MIR399* family. The image on the left shows the *Vvi-MIR399* family containing *cis*-acting elements related to three classes, including stress response (marked by a blue line), tissue-specific (marked by a green line), and progress-specific (marked by a red line). The gray, yellow, green, blue, orange, and purple squares indicated 0, 1, 2, 3, 4, and more than 4 promoters, respectively.

The distribution form and location of genes on chromosomes have important effects on gene expression and genetic linkage. By studying the location and distribution of genes on chromosomes, the functions of genes can be better understood. Based on the chromosomal localization information, the *Vvi-MIR399* family members were localized on four chromosomes (Supplementary Table S3 and Figure 3a). *Vvi-MIR399a*, *Vvi-MIR399d*, *Vvi-MIR399e*, *Vvi-MIR399f*, and *Vvi-MIR399h* were located in on Chr 10, while *Vvi-MIR399b*, *Vvi-MIR399c*, and *Vvi-MIR399i* were located on Chr 16, Chr 15, and Chr 2, respectively. *Vvi-MIR399b* and *Vvi-MIR399c* were located on different chromosomes, whereas they shared the same mature sequence. However, although the maturation sequences of *Vvi-MIR399a* and *Vvi-MIR399h* were the same, they were located in different locations on the same chromosome (Figure 3a).

To understand the possible regulatory mechanism of the *Vvi-MIR399* family members at the transcriptional level, their promoter sequences were analyzed using the online website. The *cis*-regulatory elements within 2000 bp of the *Vvi-MIR399* promoter sequence upstream of the TSS were predicted. The predicted promoter elements were classified into eight categories, including enhancer and core elements, light-related elements, defense-related elements, phytohormone-related elements, cycle and circadian rhythms, metabolism regulation, seed development, and others. As expected, the core promoter element TATA-box was observed in all the *Vvi-MIR399* promoter regions, and all the *Vvi-MIR399* gene family members contained CAAT-box, which is a common *cis*-acting element in promoter and enhancer regions (Supplementary Table S4). The main role of TATA-box was to initiate transcription precisely, while the main role of the upstream promoter element CAAT-box was to control the frequency of transcription initiation, and CAAT-box had the greatest influence on the frequency of transcription initiation. Furthermore, specific promoter elements were analyzed to understand the functional roles of the *Vvi-MIR399* family. The *Vvi-MIR399* gene family all contained more than 10 *cis*-acting elements, especially the *Vvi-MIR399g* member, which contained 32 *cis*-acting elements in its promoter (Figure 3b). The promoters of *Vvi-MIR399a*, *Vvi-MIR399b*, *Vvi-MIR399c*, *Vvi-MIR399d*, *Vvi-MIR399e*, *Vvi-MIR399f*, *Vvi-MIR399h*, and *Vvi-MIR399i* contained 17, 26, 17, 20, 12, 24, 30, and 13 *cis*-acting elements, respectively. The majority of the elements were related to ABA response, anaerobic response, drought response, GA response, light response, MeJA response, SA response, meristem-specific, protein metabolism regulation, and seed-specific regulation. Additionally, these *cis*-acting elements could be divided into three classes, including progress-specific, tissue-specific, and stress response. For plant stress response elements, 66.7% (6/9) of the *Vvi-MIR399* promoters harbored drought, GA, and SA response elements, with 44.4% (4/9) of the *Vvi-MIRR399* promoters also presenting elements involved in ABA and MeJA responses. By contrast, among the nine *Vvi-MIR399* promoters, seven, three, two, and one *Vvi-MIR399* contained anaerobic, auxin, defense and stress, and low-temperature response elements, respectively. Notably, the light-response element was presented in all *Vvi-MIR399* gene family members. These results indicate that the *Vvi-MIR399* family members might have a crucial role in responding to various phytohormones and stress, while also reflecting their potential different functions.

### 2.4. Prediction of Target Genes and Tissue-Specific Expression Analysis

**Figure 4:**
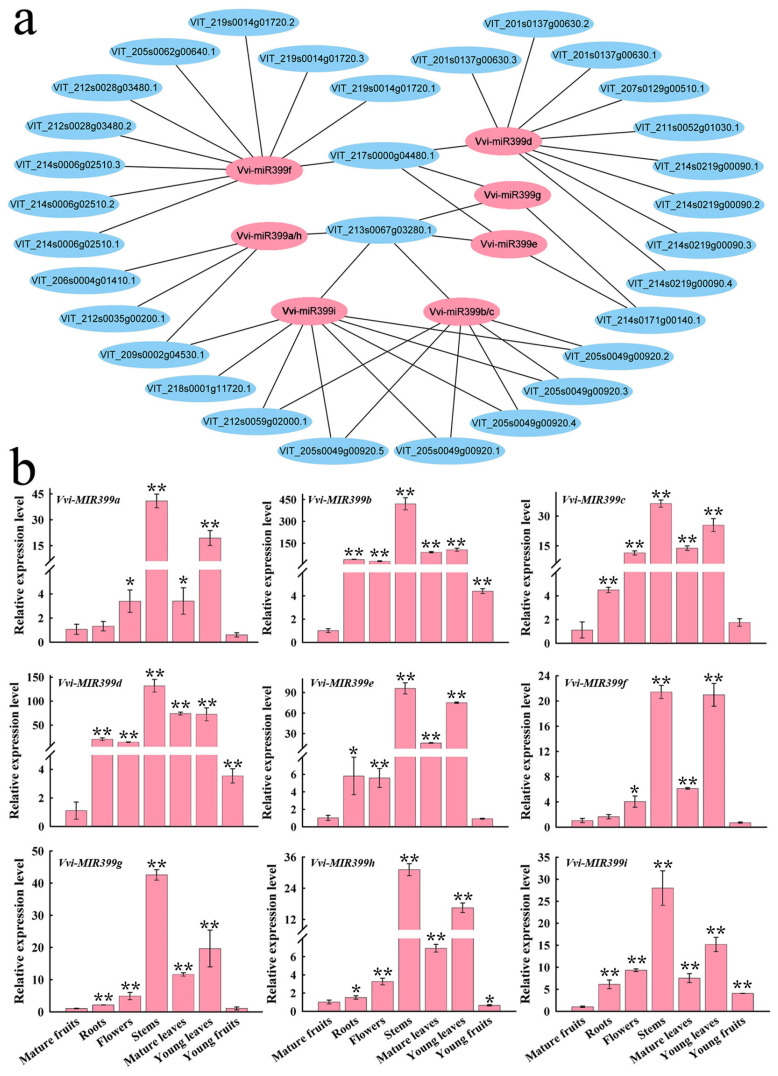
Prediction of target genes and tissue-specific expression analysis. (**a**) Prediction of interaction network Vvi-miR399 family members and their target genes. Pink and blue represent Vvi-miR399 and their target genes, respectively. (**b**) Expression analysis of grapevine *Vvi-MIR399* family members in various tissues. Error bars show the standard errors between three biological replicates. * and ** indicate significant differences at *p* ≤ 0.05 and *p* ≤ 0.01, respectively.

MiRNAs serve functions by regulating target genes. To discover the potential functions of the Vvi-miR399 family members, their target genes were predicted using the miRBase database [29]. The relationships between the Vvi-miR399 family and their predicted target genes are shown (Figure 4a). In total, 31 predicted target genes were found, and each Vvi-miR399 family member had multiple predicted target genes. Notably, some genes were targeted by multiple Vvi-miR399 family members; for example, seven Vvi-miRNA399s (miRNA399a, miRNA399b, miRNA399c, miRNA399e, miRNA399g, miRNA399h, and miRNA399i) simultaneously targeted *VIT_213s0067g03280.1*, suggesting the functional conservation and diversification of the *Vvi-MIR399* family members.

RT-qPCR was performed to further explore the expression patterns of *Vvi-MIR399* in the different tissues of grapevine (Figure 4b). The results showed that the *Vvi-MIR399* family members had varying degrees of expression in different grapevine tissues. Interestingly, the expression trends of the *Vvi-MIR399* family were consistent in different tissues, with the lowest expression level in mature and young fruits and the highest expression level in stems and young leaves. For the *Vvi-MIR399b* gene, the expression levels in stems were 420-fold higher than those in mature fruits. These results suggest that different *Vvi-MIR399* family members might play different roles in various tissues.

### 2.5. Expression Analysis of Vvi-MIR399 Family Members under Abiotic Stress

**Figure 5:**
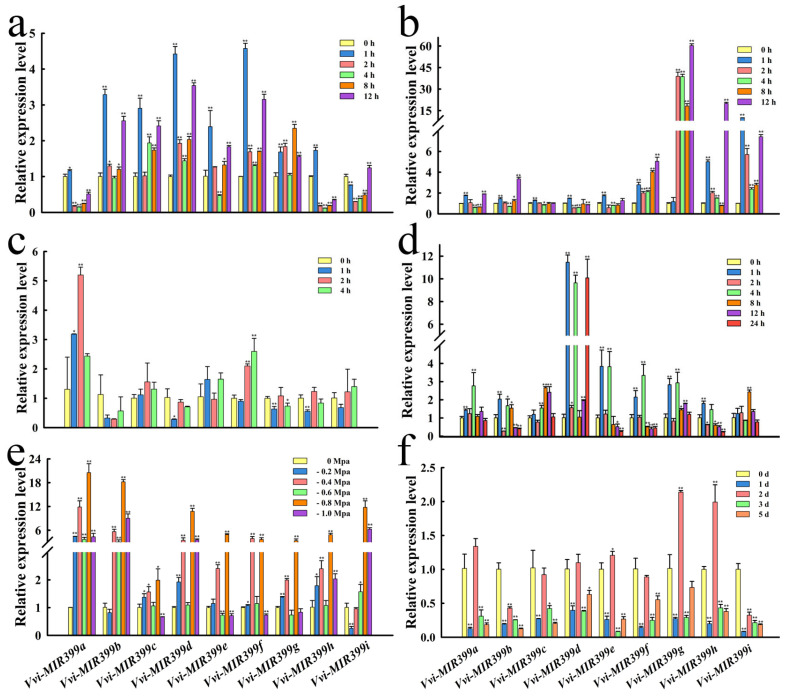
RT-qPCR analysis of the abundances of the *Vvi-MIR399* gene family members under various stress conditions. (**a**) Expression patterns of the 9 *Vvi-MIR399* genes treated with 1000 lx low light for 0, 1, 2, 4, 8, and 12 h. (**b**) Expression patterns of the 9 *Vvi-MIR399* genes treated with 30,000 lx high light for 0, 1, 2, 4, 8, and 12 h. (**c**) Expression patterns of the 9 *Vvi-MIR399* genes in seedlings under 0, 1, 2, and 4 h of heat stress at 45 °C. (**d**) Expression patterns of the 9 *Vvi-MIR399* genes in seedlings under 0, 1, 2, 4, 8, 12, and 24 h of cold stress at 2 °C. (**e**) Expression patterns of the 9 *Vvi-MIR399* genes exposed to drought stress at 0, −0.2, −0.4, −0.6, −0.8, and −1.0 Mpa. (**f**) Expression patterns of the 9 *Vvi-MIR399* genes after exposure to 300 mM NaCl solution for 0, 1, 2, 3, and 5 h. All treatments were conducted with leaves as the tissue material and 0 h as the control. Error bars show the standard errors between three biological replicates. * and ** indicate significant differences at *p* ≤ 0.05 and *p* ≤ 0.01, respectively.

In order to explore the function and effect of *Vvi-MIR399s* in grapevine, the expression patterns of *Vvi-MIR399s* under low light, strong light, high temperature, cold, drought, and salt stress were investigated (Figure 5). When grapevine plants were exposed to low light stress, *Vvi-MIR399a*, *Vvi-MIR399g*, and *Vvi-MIR399h* showed similar expression patterns, with their lowest levels at 4 h. The expression levels of *Vvi-MIR399b*, *Vvi-MIR399c*, *Vvi-MIR399d*, *Vvi-MIR399e*, and *Vvi-MIR399f* were upregulated and increased by more than 2.90-fold at 1 h compared with the control samples at 0 h, followed by a sharp decrease at 4 h and a further increase at 12 h. All nine *Vvi-MIR399s* showed a rapid decrease in abundance at 4 h (Figure 5a). Therefore, the *Vvi-MIR399* gene family members were potentially involved in low light stress during an early stage. The expression level of *Vvi-MIR399g* increased sharply after 1h with the extension of high light stress time, while the transcripts of *Vvi-MIR399a*, *Vvi-MIR399c*, *Vvi-MIR399d*, and *Vvi-MIR399e* were barely induced during high light stress. After 12 h of high light treatment, the expression levels of *Vvi-MIR399b*, *Vvi-MIR399f*, *Vvi-MIR399g*, and *Vvi-MIR399h* increased significantly by 3.36-, 5.05-, 60.28-, and 20.03-fold, respectively, indicating that they might respond to high light stress at a later stage (Figure 5b). When grapevine plants were exposed to heat stress, the expression of *Vvi-MIR399b*, *Vvi-MIR399d*, *Vvi-MIR399g*, *Vvi-MIR399h*, and *Vvi-MIR399i* were promptly decreased at 1 h after treatment, indicating that they might be relatively sensitive to heat stress. The abundances of *Vvi-MIR399a* and *Vvi-MIR399f* were upregulated at 2 h and 4 h and increased by 5.20- and 2.59-fold, respectively, compared with the control samples at 0 h (Figure 5c). Under cold stress, the expressions of the *Vvi-MIR399d*, *Vvi-MIR399e*, *Vvi-MIR399f*, *Vvi-MIR399g*, and *Vvi-MIR399h* genes were rapidly increased at 1 h, suggesting that they might be responsive to cold stress at an early stage. In particular, the expression of *Vvi-MIR399d* reached its maximum levels at 1 h, 4 h, and 24 h under cold stress, with 11.46-, 9.64-, and 10.08-fold changes, respectively, compared with the control (Figure 5d). In grapevine exposed to drought stress, the nine *Vvi-MIR399* gene family members showed similar accumulation patterns, reaching the maximum accumulation at −0.8 MPa, followed by a sharp decrease at −1.0 MPa (Figure 5e). When grapevine was subjected to salt stress, the nine *Vvi-MIR399s* first decreased, then increased, and then decreased and remained at a low level. The *Vvi-MIR399a*, *Vvi-MIR399d*, *Vvi-MIR399e*, *Vvi-MIR399g*, and *Vvi-MIR399h* genes showed similar accumulation patterns, with their levels reaching their peaks at 2 d and their maximal fold changes ranging from 1.10 to 2.12. These results suggest that the *Vvi-MIR399* gene family members might respond to salt stress at an early stage (Figure 5f).

### 2.6. Expression Patterns of Target Genes in Response to Abiotic Stresses

**Figure 6:**
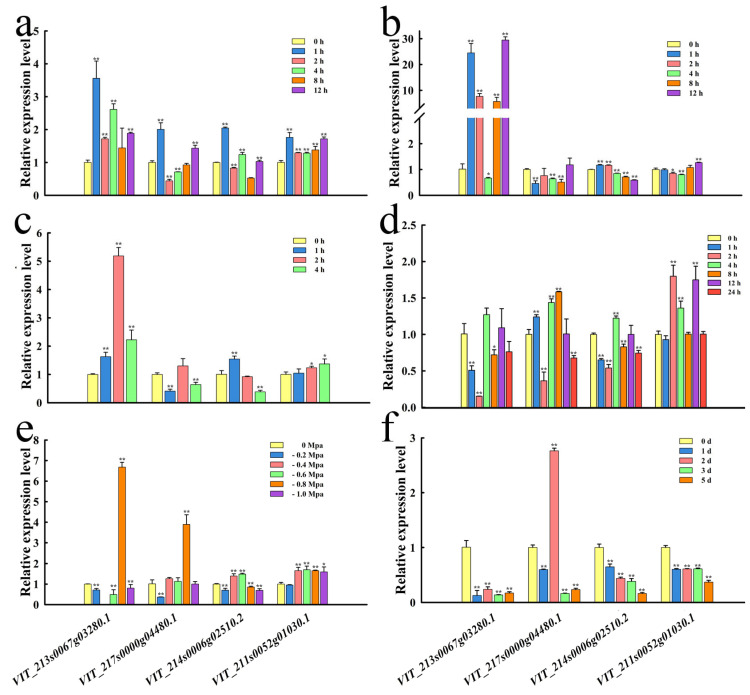
Expression analysis of the four Vvi-miR399 target genes in response to abiotic stresses. (**a**) Expression patterns of the four Vvi-miR399 target genes treated with 1000 lx low light for 0, 1, 2, 4, 8, and 12 h. (**b**) Expression patterns of the four Vvi-miR399 target genes treated with 30,000 lx high light for 0, 1, 2, 4, 8, and 12 h. (**c**) Expression patterns of the four Vvi-miR399 target genes in seedlings under 0, 1, 2, and 4 h of heat stress at 45 °C. (**d**) Expression patterns of the four Vvi-miR399 target genes in seedlings under 0, 1, 2, 4, 8, 12, and 24 h of cold stress at 2 °C. (**e**) Expression patterns of the four Vvi-miR399 target genes exposed to drought stress at 0, −0.2, −0.4, −0.6, −0.8, and −1.0 Mpa. (**f**) Expression patterns of the four Vvi-miR399 target genes after exposure to 300 mM NaCl solution for 0, 1, 2, 3, and 5 h. All treatments were conducted using leaves as tissue material and 0 h as the control. Error bars show the standard errors between three biological replicates. * and ** indicate significant differences at *p* ≤ 0.05 and *p* ≤ 0.01, respectively.

To explore the regulatory function of miR399s in grapevine, the expression patterns of four target genes with a high degree of confidence were also predicted and analyzed using RT-qPCR in response to abiotic stresses (Figure 6). The four genes were promptly increased to their peaks in transcript accumulation at 1 h after low light stress, and their fold changes ranged from 1.76 to 3.56, respectively, compared with the control, indicating that they might be responsive to low light stress at an early stage (Figure 6a). After high light stress, the transcription levels in *VIT_214s0006g02510.2* gradually increased by 1.17- and 1.16-fold at 1 and 2 h compared with the 0 h control and then gradually decreased until 12 h. The abundance of *VIT_213s0067g03280.1* also increased 24.49-fold at first, then decreased, but then rapidly increased 29.46-fold at 12 h (Figure 6b). *VIT_211s0052g01030.1* showed a gradual increase by 1.37-fold in transcript accumulation with prolonged stress, while *VIT_213s0067g03280.1* and *VIT_214s0006g02510.2* exhibited slight decreases at late stages of heat stress (Figure 6c). In the context of cold stress, *VIT_213s0067g03280.1*, *VIT_214s0006g02510.2*, and *VIT_217s0000g04480.1* showed similar trends in their abundances, with sharp decreases and reaching their lowest levels at 2 h and modest increases in their levels at 4 and 12 h. The Vvi-miR399 family members might thus promote the accumulation of their target transcripts and play a positive role in stress adaptation (Figure 6d). *VIT_213s0067g03280.1* and *VIT_217s0000g04480.1* reached their maximum levels at −0.8 MPa drought stress with 6.68- and 3.89-fold changes, respectively, compared with the control (Figure 6e). Compared with the control, the expressions of *VIT_213s0067g03280.1*, *VIT_214s0006g02510.2*, and *VIT_211s0052g01030.1* were obviously decreased by salt stress. In contrast, the transcript accumulations of *VIT_217s0000g04480.1* were increased by 2.76-fold at 2 d after treatment (Figure 6f).

### 2.7. Overexpression of Vvi-MIR399 Enhanced the Drought Tolerance in Arabidopsis

**Figure 7:**
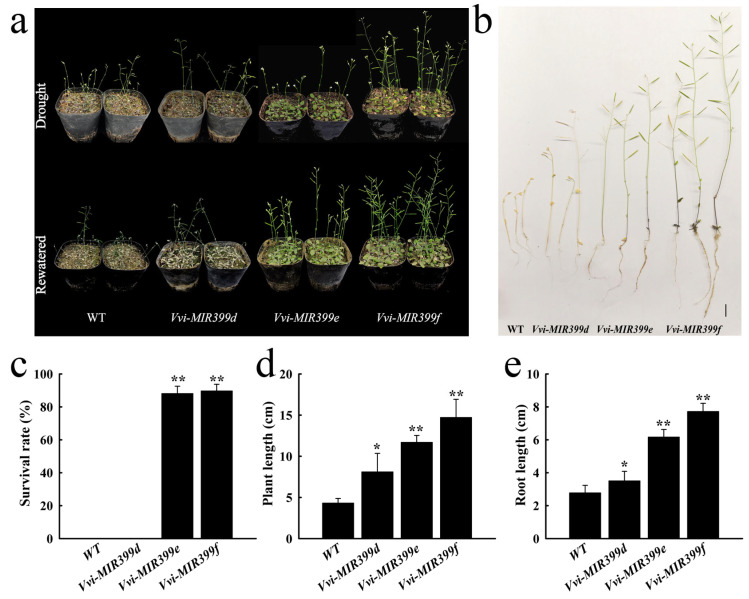
Phenotypic identification and analysis of three *Vvi-MIR399*-overexpressing plants (*Vvi-MIR399d*, *Vvi-MIR399e*, and *Vvi-MIR399f*) under drought stress. (**a**) Growth of *Vvi-MIR399d-f*-overexpressing plants under drought stress and water stress recovery. (**b**) Whole plant phenotype (**c**–**e**) of *Vvi-MIR399d-f-*overexpressing plants under water stress recovery water after drought stress. (**c**–**e**) Survival rate, plant length, and root length of *Vvi-MIR399d-f*-overexpressing plants. * and ** indicate significant differences at *p* ≤ 0.05 and *p* ≤ 0.01, respectively.

It is known that the secondary structural determinants of precursors, such as the stem–loops/bulges and sequences surrounding the miRNA/miRNA* region, have an important influence on miRNA processing [41,42]. The different miRNA399 precursors of grapevine show variations in their structures as well as differences in the sequences in the stem parts of their precursors (Figure 2). Therefore, to investigate the biological function of *MIR399* in response to drought stress in grapevine according to the upregulation of the *Vvi-MIR399* family in grapevine drought stress, three *Vvi-MIR399* family members (*Vvi-MIR399d*, *Vvi-MIR399e*, and *Vvi-MIR399f*) were overexpressed in *Arabidopsis*. Ten-day-old seedlings with consistent growth were grown under drought stress for 30 days: *Vvi-MIR399e*- and *Vvi-MIR399f*-overexpressed materials grew normally, whereas the leaves of *Vvi-MIR399d* were dry and the plants grew slowly (Figure 7a). When these transgenic *Arabidopsis thaliana* seedlings recovered after water stress, the seeding survival rates of *Vvi-MIR399e* and *Vvi-MIR399f* were 88.2% and 89.8%, respectively; i.e., significantly higher than those of WT and *Vvi-MIR399d* (Figure 7c). However, the plant lengths of these transgenic plants were significantly greater than that of WT (Figure 7d). In order to more directly observe the growth of transgenic plants under drought stress and control the root system, the roots were cleaned and observed. The results showed that the root lengths of the *Vvi-MIR399d-*, *Vvi-MIR399e-*, and *Vvi-MIR399f-*overexpressed materials were significantly greater than that of WT (Figure 7b, e). *Vvi-MIR399s* played an important role in drought stress, of which *Vvi-MIR399e* and *Vvi-MIR399f* were the most drought tolerant.

## 3. Discussion

### 3.1. Evolutionary Characteristics of the MIR399 Family

MiR399 acts as a conserved miRNA to be implicated in a wide range of cellular and physiological processes in plants [43]. Based on data from the miRBase, most plant species harbor multiple miR399s. It is known that the existence of multiple copies of miRNA genes within plant species provides one possibility for functional redundancy and specificity [44]. Plant miRNAs are thought to mediate almost all plant cellular and metabolic processes via the regulation of posttranscriptional gene silencing [6]. Conserved miRNAs might play crucial roles in regulating fundamentally important biological processes. A hallmark of RNA silencing was a class of approximately 22-nucleotide RNAs processed from double-stranded RNA precursors by Dicer. Accurate processing by Dicer was crucial for the functionality of miRNAs. MiRNAs can regulate target mRNA by disrupting its stability and inhibiting its translation. In this study, the majority of the Vvi-miR399 sequences are highly conserved between 2 and 20 nt, and the diversity lies in their ends (Figure 2c). The 5′-end recognition by Dicer is important for the precise and effective biogenesis of miRNAs [45]. The 5′ variability might have a bearing on the association with different AGOs [46]. Eight mature sequence variants of Vvi-miR399 had ‘T’ at the 5′ terminal nucleotide, suggesting that they primarily load on AGO1. In contrast, one Vvi-miR399 mature variant had ‘C’ at the 5′ terminal base (including Vvi-miR399i), suggesting that it is mainly recruited into AGO5 followed by AGO1. The 3′-end was displayed as a key determinant regulating miRNA activity via 3′-remodeling and/or degradation [47]. Therefore, the diversity and evolutionary conservation of the miR399 gene family are expected to be elucidated to further understand their regulatory roles in plant development and/or stress resistance.

It was proposed that conserved miRNA families have been both conserved and diversified during miRNA evolution [44]. In this study, our data indicated that grapevine miR399s showed evolutionary conservation. We identified nine *MIR399* genes in grapevine. In rice, eleven *MIR399* exist [43], and in dicot *Arabidopsis* [48], tomato [49], and sweet orange [50], six, two, and six *MIR399* are present, respectively (Figure 2d). The multiple miR399s likely diverged from a common ancestor, as reflected by the evolutionary diversity of the miR399 precursor sequences and most of the *Vvi-MIR399* gene family being clustered with the *MIR339* genes from dicot plants. According to the phylogenetic tree, Vvi-miR399a, Vvi-miR399b, Vvi-miR399c, Vvi-miR399h, and Vvi-miR399i were all clustered together, signifying a common ancestry and similar course of evolution. Vvi-miR399g and Vvi-miR399d formed a branch with Vvi-miR399e, indicating that the Vvi-miR399s were relatively conservative in their evolution and function. Notably, Vvi-miR399f formed a separate branch (Figure 2e). These observations suggest that the majority of the Vvi-miR399 members had a close evolutionary relationship, while exceptions also existed, which might be related to differences in function. In addition, *Vvi-MIR399a* and *Vvi-MIR399h* had the same mature sequence and were located on the same chromosome, indicating that Vvi-miR399a and Vvi-miR399h might have the same function in grapevines (Figure 3a). Numerous previous studies showed that gene expression is controlled by upstream elements such as the promoter [51,52]. Promoters are important parts of genes; their main function is to regulate the initiation time and degree of gene expression (transcription). However, promoters do not control gene activity by themselves but by binding to transcription factors. In addition, analysis of the *Vvi-MIR399* promoters showed that *Vvi-MIR399* exerted multiple functions. TATA-box and CAAT-box, which were required for transcriptional initiation, and light-response elements were most enriched in the *Vvi-MIR399* promoter region, suggesting that these *cis*-elements were fundamental to the expression of the *Vvi-MIR399* family genes (Figure 3b). Drought-response-related elements were found in the promoters of *Vvi-MIR399b*, *Vvi-MIR399c*, *Vvi-MIR399f*, *Vvi-MIR399g*, *Vvi-MIR399h*, and *Vvi-MIR399i*, suggesting that they might be involved in the regulation network of drought stress signals. Low temperature-response-related elements were found only in the promoter of *Vvi-MIR399b*, which might be involved in the low-temperature stress signal regulation network. Moreover, defense-related elements (TC-rich repeats, LTR, ARE, and MBS) and phytohormone-related elements (TGA-element, TATC-box, TCA-element, ABRE, CGTCA-motif, TGACG-motif, GARE-motif, and P-box) were also present in some *Vvi-MIR399* family members, and they regulated the precise initiation and transcriptional efficiency of gene transcription by binding to transcription factors. These results suggest that the *Vvi-MIR399s* might have a crucial role in responding to various phytohormones and stress, while also reflecting their potential different functions (Supplementary Table S4). Numerous studies showed that miRNAs function as negative regulators for the expression of target genes, playing a crucial role in regulating the growth, development, and stress response of plants. The prediction of Vvi-miR399 target genes showed that most of the Vvi-miR399s had different target genes (Figure 4a). Interestingly, it was found that one gene (*VIT_213s0067g03280.1*) could be targeted by more than one miRNA. *VIT_213s0067g03280.1* harbored a highly conserved miR399 binding site in grapevine, and miR399s might have redundancies in their functions. These results suggest that the miR399s family in grapevine have different functions.

### 3.2. The MIR399 Family May Participate in Plant Growth and Development

MiR399 was shown to be involved in the growth and development of plants. For example, the silencing of miR399 expression can disrupt the peanut meristem and anther development of citrus [38], and overexpression of miR399 can affect the fruit quality of strawberries [53]. In addition, miR399 is involved in color formation in tomato and apple fruits [54,55]. Overexpressing miR399 in maize (*Zea mays* L.) was associated with premature senescence after pollination [56]. Enhanced freezing tolerance was observed for tae-miR399-overexpressing *Arabidopsis* lines [40]. In *Arabidopsis*, miR399 generated in shoots serves as a long-distance signal that represses *PHO2* expression in roots under Pi-limiting conditions, resulting in the activation of Pi uptake and translocation [57]. The results of real-time PCR further showed that *Vvi-MIR399s* show strong expression in stems, mature leaves, and young leaves, suggesting its role in stem and leaf development (Figure 4b). Meanwhile, the expression degrees of the *Vvi-MIR399* were different in different tissues of grapevine. The expression of *Vvi-MIR399b* in stems was 420-fold higher than that in mature fruits, which indicated its developmental specificity in stems. These results indicate that *Vvi-MIR399* family members were expressed in different tissues of grapevine, and the expression trend was consistent, highlighting the developmental specificity.

### 3.3. The MIR399 Family May Respond to Abiotic Stress in Plants

Previous studies showed that plants respond to abiotic stress by modulating gene expression at post-transcriptional levels via miRNAs [48]. Abiotic stress affects various physiological processes of plant development. Exposure to different abiotic stresses can lead to similar responses in plants. Moreover, different kinds of stresses can trigger responses by the induction of similar types of miRNAs [58]. This indicates that plants share common signaling pathways that act in different abiotic stress responses. After exposure to stress treatments that influenced plant growth and developmental processes, the relevant miRNAs were either down-regulated or up-regulated [59].

Light intensity plays a central role in obtaining energy for plants to survive [60]. Low light stress is a major abiotic stress for the grape industry and can significantly affect plant growth and development [61], especially during the rainy season. The upregulation of *Vvi-MIR399b*, *Vvi-MIR399c*, *Vvi-MIR399d*, *Vvi-MIR399e*, and *Vvi-MIR399f* under low light stress indicate that they might act as positive regulators of low light stress (Figure 5a). Plants have evolved sophisticated photoreceptors to control many biochemical and physiological parameters by adapting to different light environments in order to increase their photosynthetic and metabolic performance [62,63]. Light intensity plays a major role in plant germination, cell division, photosynthesis, leaf proliferation, and expansion [64,65,66]. Low light stress reduces inflorescences, resulted in smaller and thinner leaves, shortened flowering periods, and delayed flower bud differentiation [67,68,69]. When grapevine plants were exposed to high light stress, the expression of *Vvi-MIR399s* (*Vvi-MIR399b*, *Vvi-MIR399f*, *Vvi-MIR399g*, and *Vvi-MIR399h*) promptly increased at 12 h after treatment, indicating that they might be relatively sensitive to high light stress at a late stage. The *Vvi-MIR399* family members harbored light-related elements in promoter regions, which might be involved in light responsiveness and affect the growth and development of grapevines (Figure 3b). The G-box binding factor acts as a positive regulator of lateral root formation, and it differentially regulates the expression of light-inducible genes [70]. AE-box and ATCT-motif are present within the 130-bp fragment of the D2-AtPolλ promoter and regulate the light-mediated activity of the promoter via binding of specific *trans*-acting factors [71]. Wide- and narrow-leaf plants have distinct frequencies of GATA-motif elements in chloroplast genomes [72]. Indeed, *Vvi-MIR399f* and *Vvi-MIR399g* were promptly increased by low and high light stress, implying that Vvi-miR399f and Vvi-miR399g can possibly function as modulators of light-induced signaling pathways.

MiR399 is a kind of miRNA that is sensitive to environmental temperature [73], and miR399b-overexpressing plants and a loss-of-function allele of *PHO2* (*pho2*) exhibited an early flowering phenotype only at normal temperature (23 °C). Interestingly, their flowering time at a lower temperature (16 °C) was similar to that of WT, the expression of *TWIN SISTER OF FT* (*TSF*) was increased in miR399b-overexpressing plants and *pho2* mutants at 23 °C. In this study, the downregulation of *Vvi-MIR399d* and the concomitant upregulation of its target gene encoding on *VIT_211s0052g01030.1* showed that *Vvi-MIR399d* plays a critical role in regulating heat stress responses in this species. When grapevine plants were exposed to heat stress, the expression of *Vvi-MIR399s* (*Vvi-MIR399b*, *Vvi-MIR399d*, *Vvi-MIR399g*, *Vvi-MIR399h*, and *Vvi-MIR399i*) were promptly decreased at 1 h after treatment, indicating that they might be relatively sensitive to heat stress at an early stage. Under heat stress, *Vvi-MIR399a* and *Vvi-MIR399f* were up-regulated at 2h and 4 h (Figure 5c), while the target genes *VIT_213s0067g03280.1* of *Vvi-MIR399a* and *VIT_217s0000g04480.1* of *Vvi-MIR399f* were up-regulated at 2 h and down-regulated at 4 h (Figure 6c). These results suggested that *Vvi-MIR399s* mediated the silencing of target genes was under sophisticated regulation. Under freezing stress of transgenic *Arabidopsis* plants, overexpression of tae-miR399 ultimately decreased the expression of *AtUBC24*, inhibiting the degradation of *AtICE1*, which increased the expression of genes involved in the CBF signaling pathway and starch metabolism and promoted the activities of antioxidant enzymes [40]. In *Saccharomyces cerevisiae*, only overexpression of both *GTR1* and *PHO84* (*VIT_213s0067g03280.1*) could restore cold tolerance to an extent equivalent to the genomic fragment, indicating that the original tolerance was due to an additive effect of *GTR1* and *PHO84* [74]. *Vvi-MIR399b* harbored the LTR *cis*-acting element in promoter regions, which can be involved in low-temperature responsiveness (Figure 3b). Indeed, the abundance of *Vvi-MIR399b* under cold stress reached its lowest levels at 2 h and a modest increase in their levels at 1, 4, and 8 h. Similarly, the accumulation of the Vvi-miR399b target gene *VIT_213s0067g03280.1* under cold stress decreased sharply within 2 h and then gradually increased (Figure 6d). After 1 h of cold stress, the expression of some *Vvi-MIR399s* (*Vvi-MIR399d*, *Vvi-MIR399e*, *Vvi-MIR399f*, *Vvi-MIR399g*, and *Vvi-MIR399h*) increased rapidly (Figure 5d), implying that Vvi-miR399b might respond to cold stress at an early stage.

In addition, miR399 was demonstrated to alter in abundance in response to salt stress. MiR399 abundance was altered by the cultivation of Col-0 seedlings for a 7-day period in the presence of 150 mM NaCl [75]. In contrast to the *MIM399* transformant line, the rosette of the *MIR399* plant line was slightly reduced in size compared with Col-0 rosettes. Furthermore, in the distal tips of mature rosette leaves of the *MIR399* plant, areas of chlorotic and necrotic tissue formed. In addition to this vegetative phenotype, the inflorescence stem and its siliques were a pale green to yellow color in contrast to the uniform, healthy green coloration of the reproductive organs of Col-0 plants [76]. The roots from plants overexpressing miR399a exhibited increased sensitivity to salt stress when treated with 75 mM NaCl. Primary root growth in the miR399a-overexpressing roots was reduced by 40%, and the lateral root number per hairy root was decreased by up to 70% under salt stress conditions [77]. In addition, *PMI2* (*plastid movement impaired*) was reported to share sequence and structural similarity with *PMI15 (VIT_214s0006g02510.2)*, another unknown protein in *Arabidopsis* that, when mutated, causes a defect in chloroplast avoidance under high light intensities [78]. The expressions of *VIT_217s0000g04480.1* from the *AP2*/*ERF* family were obviously increased by salt stress. In maize, *AP2*/*ERF* family member *ZmEREB20* was reported to positively regulate salt tolerance through molecular mechanisms associated with hormone signaling, ROS scavenging, and root-hair plasticity [79]. In our study, the abundances of *Vvi-MIR399b*, *Vvi-MIR399c*, *Vvi-MIR399f*, and *Vvi-MIR399i* decreased under salt stress (Figure 5f), and the expressions of the Vvi-miR399b, Vvi-miR399c, and Vvi-miR399i target gene *VIT_213s0067g03280.1* and the Vvi-miR399f target gene *VIT_214s0006g02510.2* were also down-regulated (Figure 6f). Thus, Vvi-miR399b, Vvi-miR399c, Vvi-miR399f, and Vvi-miR399i might act as negative regulators of salt stress.

The expression levels of members of the *Arabidopsis thaliana* miR399 family under drought stress varied by different fold levels [80], and the down-regulation of ssp-miR399-seq 1 under drought stress in both sugarcane cultivars likely led to increased pyrophosphatase levels [81]. Drought stress treatment of *Arabidopsis thaliana* seedlings increased miR399 abundances by 2.7-fold [75]. MiR399 was up-regulated 13-fold in tobacco seedlings after exposure to 7.5% PEG [82]. In our study, the levels of seven *Vvi-MIR399* family members (*Vvi-MIR399a*, *Vvi-MIR399c*, *Vvi-MIR399d*, *Vvi-MIR399e*, *Vvi-MIR399f*, *Vvi-MIR399*g, and *Vvi-MIR399h*) were significantly induced at −0.4 MPa and −0.8 MPa. However, *Vvi-MIR399b* and *Vvi-MIR399i* decreased at −0.2 MPa and had only one peak (Figure 5e). In addition, we found that *Arabidopsis thaliana* overexpressing *Vvi-MIR399e* and *Vvi-MIR399f* were more drought tolerant than those overexpressing *Vvi-MIR399d*. The survival rate of *Vvi-MIR399e* and *Vvi-MIR399f* after water stress recovery was reported to be significantly higher than those of WT and *Vvi-MIR399d*, implying that *Vvi-MIR399e* and *Vvi-MIR399f* could possibly function as modulators of drought-induced signaling pathways. We presume that different members of the Vvi-miR399 gene family exhibit distinct expression patterns and form a complex regulatory network under drought stress.

## 4. Materials and Methods

### 4.1. Plant Materials and Stress Treatments

The tissue-cultured seedlings of the ‘Thompson seedless’ grape variety were maintained in Murashige and Skoog (MS) medium with 0.1 mg/L IAA, which was sub-cultured every 40 d for light intensity treatments. All grapevines used for light intensities treatments were grown at 25 °C under a 16-h-light/8-h-dark photoperiod. Grapevine seedlings were exposed to 1000 lx and 30,000 lx light intensities for 0, 1, 2, 4, 8, and 12 h, respectively.

One-year-old plants from the grapevine cultivar ‘Muscat Hamburg’, grown in a growth chamber, were used for heat, cold, drought, and salt treatments at 45 °C for 0, 1, 2, and 4 h for heat treatments and at 2 °C for 0, 1, 2, 4, 8, 12, and 24 h for cold treatments. For drought treatment, plants were transferred to soil with soil water potential of 0, −0.2, −0.4, −0.6, −0.8, and −1.0 Mpa. Plants were watered with a 300 mM NaCl solution for 0, 1, 2, 3, and 5 h for salt treatments. For all treatments, the 0 h treatment served as the control. The leaves were collected and immediately frozen in liquid nitrogen for further experiments.

### 4.2. Identification of the miR399 Family in Grapevine

The precursor sequences and mature sequences of miR399 family members in the plants were obtained from the miRbase database (https://www.mirbase.org, accessed on 26 August 2023) (Supplementary Table S1) [29].

### 4.3. Experimental Verifications of the Vvi-MIR399 Family Members

The primers were designed using the Primer-Blast tools in NCBI (www.ncbi.nlm.nih.gov, accessed on 26 August 2023) (Supplementary Table S2). All *Vvi-MIR399* family sequences were amplified and cloned into the pEASY^®^-Blunt Cloning vector using the pEASY^®^-Blunt Cloning Kit (TIANGEN, Beijing, China). Cloned precursors were further confirmed by Sanger sequencing.

### 4.4. Structure Prediction of the Vvi-miR399 Precursors

The precursors of *Vvi-MIR399s* were input on the RNAfoldWebServer website (http://rna.tbi.univie.ac.at/cgi-bin/RNAWebSuite/RNAfold.cgi, accessed on 27 August 2023), the secondary structures of the *Vvi-MIR399* family member precursors were predicted, and the parameters were set as default.

### 4.5. Phylogenetic Analyses of the Vvi-MIR399 Family Members

The precursor and mature sequences of the *Vvi-MIR399s* were entered on MEGA 11 software. Multiple alignments of all *Vvi-MIR399s* sequences and phylograms were drawn using the ClustalX program with the Maximum Likelihood Estimate method in MEGA 11 with a bootstrap analysis of 1000 replicates.

### 4.6. Conservation Analysis of the Vvi-miR399 Family Members

Multiple alignment of the Vvi-miR399 family members was conducted using DNAMAN software v6.0.3.99, and the results were submitted to WebLogo (http://weblogo.berkeley.edu, accessed on 27 August 2023) for base preference analysis using default parameters.

### 4.7. Chromosome Localization of the Vvi-MIR399 Family

To determine the locations of the *Vvi-MIR399* family members on grapevine chromosomes, a search was conducted using the miRBase database (https://www.mirbase.org, accessed on 28 August 2023) (Supplementary Table S3), and the results were imported to MG2C_v2.1 (http://mg2c.iask.in/mg2c_v2.1, accessed on 28 August 2023) for visualization.

### 4.8. Regulatory Element Prediction on the Promoter Regions of the Vvi-MIR399 Family

The website PlantCARE (http://bioinformatics.psb.ugent.be/webtools/plantcare/html, accessed on 29 August 2023) was employed to predict the *cis*-acting regulatory elements on the promoters (2000 bp upstream of the precursor sequence) of the *Vvi-MIR399* gene family members. The number, distribution, and classification of elements were analyzed (Supplementary Table S4).

### 4.9. Prediction of Vvi-miR399-Targeted Genes

The target genes of the Vvi-miR399s were predicted using psRNATarget [83]. The psRNATarget parameter settings were set as default with a maximum exception value of 3. The relationship between Vvi-miR399 and the predicted target genes were visualized using Cytoscape [84] (version 3.9.1, created by Ideker et al., San Diego, CA, USA).

### 4.10. Expression Analysis of the Vvi-MIR399 Family and Target Genes in Grapevine

The total RNA was isolated from grapevine under different tissues and different stress treatments using a modified CTAB method [85] and then transcribed to cDNA, and the results were used as templates for RT-qPCR. The RT-qPCR was performed on a CFX Connect Real-Time Detection System (Bio Rad, Hercules, CA, USA). The program settings were as follows: 95 °C for 150 s, followed by 40 cycles of 95 °C for 5 s and 60 °C for 30 s. The relative expression levels were analyzed using the 2^−∆∆CT^ method [86]. Actin was used as an internal control. All RT-qPCRs were performed in triplicate. Primer sequences are listed in Supplementary Tables S2 and S5.

### 4.11. Vector Construction

*Vvi-MIR399d*, *Vvi-MIR399e*, and *Vvi-MIR399f* were constructed onto the hyperexpression vector PHB. The object sequence is shown in Supplementary Table S6. The constructed plasmids were transformed into *Agrobacterium tumefaciformis* GV3101 and incubated on LB medium supplemented with rifampicin (Rif) and kanamycin (Kan) at 28 °C for 2d. Single clones were incubated in LB liquid medium containing Rif and Kan at 200 rpm and 28 °C for 10–12 h. Positive clones were added to equal volumes of 50% glycerol and stored at −80 °C.

### 4.12. Arabidopsis Transformation

The transformation of *Arabidopsis* was performed according to the floral dip method [87]. Glufosinate ammonium 10% solution (dilute 1000×) (Sangon Biotech, Shanghai, China) was used to screen transgenic seedlings. Six biological replicates were selected for each sample as plant length and primary root length measurements.

## 5. Conclusions

In this study, nine mature members and nine precursor members of the grapevine miR399 family were identified. The precursor sequence of miR399 was highly diverse, while the mature sequence was highly conserved. Promoter analysis identified multiple stress-response elements such as ABA, drought, and low temperature responses, in which the description and number of stress-response elements depended on the specific member of the *Vvi-MIR399* family, thereby determining the functional specificity of each member. Moreover, the difference in the transcript level of the *Vvi-MIR399* member confirmed the functional diversity under various abiotic stresses. Furthermore, transgenic *Vvi-MIR399e* and *Vvi-MIR399f* plants were more drought tolerant than *Vvi-MIR399d* plants under drought stress, while transgenic plants *Vvi-MIR399d* and WT had no drought tolerance. This study not only increases the understanding of the grapevine *MIR399* family but also increases their regulation and functional differentiation in abiotic stress, as well as their potential applications in crop improvement.

## Data Availability

All data supporting the findings of this study are available within this article and the Supplementary Materials published online.

## Supplementary Materials

The following supporting information can be downloaded at: https://www.mdpi.com/article/10.3390/ijms25052979/s1.
