# Supplementary material for: Identification and Analysis of the MIR399 Gene Family in Grapevine Reveal Their Potential Functions in Abiotic Stress
Source: Int J Mol Sci. 2024 Mar 4;25(5):2979. doi: 10.3390/ijms25052979 (PMC10931670; doi:10.3390/ijms25052979)
Supplement: Supplementary file 1 [file ijms-25-02979-s001.zip › Supplemental Figure.pdf]

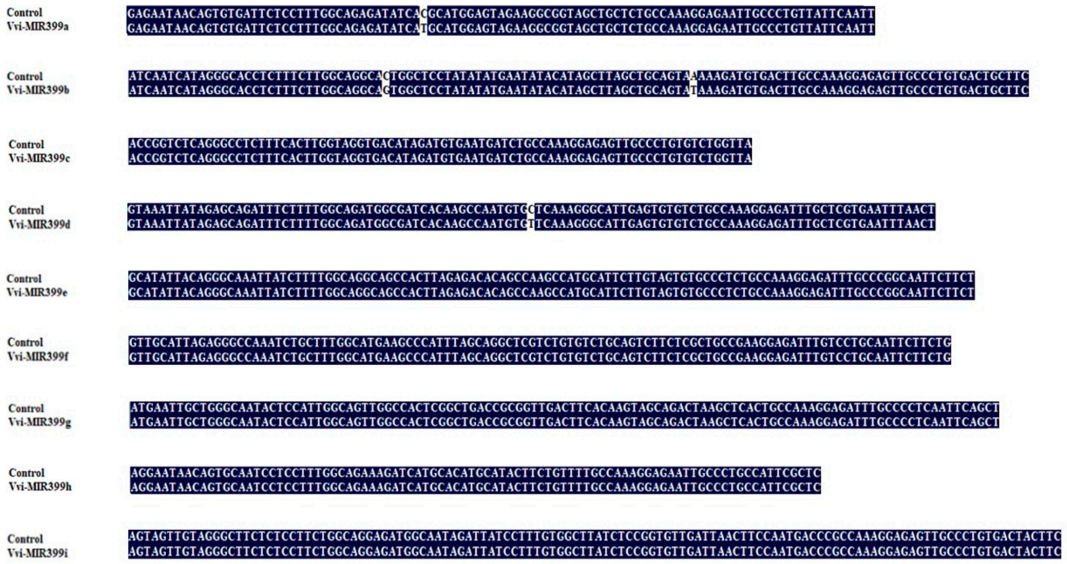

Figure S1 *MIR399* gene family sequence alignment in 'Thompson Seedless' grapevine and the miRBase database. Control and *Vvi-MIR399* represent miRBase database and 'Thompson Seedless' grapevine, respectively.
